# MHz repetition rate femtosecond radially polarized vortex laser direct writing Yb:CaF_2_ waveguide laser operating in continuous-wave and pulsed regimes

**DOI:** 10.1515/nanoph-2023-0396

**Published:** 2023-12-25

**Authors:** Kaixin Liu, Yue Dong, Zihao Zhang, Xinghao Duan, Ruohao Guo, Zhongjun Zhai, Junli Wang

**Affiliations:** School of Physics, Xidian University, Xi’An 710071, P.R. China; State Key Laboratory of Crystal Materials, Shandong University, Jinan 250100, P.R. China

**Keywords:** MHz repetition rate, femtosecond radially polarized vortex laser, transition metal sulfides, calcium fluoride crystal, waveguide laser

## Abstract

In this paper, we report the use of femtosecond radially polarized vortex laser with MHz repetition rate for direct writing of cladding waveguides (WGs) and realization of waveguide laser oscillations in ytterbium-doped calcium fluoride crystal. The negative refractive index modification in Yb:CaF_2_ crystal is fabricated by the homemade all-fiber laser amplifier. At 976 nm pump wavelength, these Yb:CaF_2_ WGs can achieve continuous-wave (CW) laser oscillation. The length of resonant cavity is 4 mm, and the minimum laser threshold is 116 mW, corresponding to the propagation loss of 0.85 dB/cm, the center wavelength of 1045.2 nm, and the maximum output power of 91 mW. In addition, a saturable absorber mirror (SAM) was prepared by depositing ReS_0.8_Se_1.2_ on the reflective surface of a dichroic mirror to realize Q-switched waveguide laser output. The output Q-switched pulses with a tunable repetition rate in the range of 125–692.5 kHz, and the shortest pulse duration is 513 ns.

## 1 Introduction

Since lasers entered the femtosecond era, their properties, such as ultra-short pulse duration and ultra-high peak power, have revolutionized laser micro-machining technology and opened up a new way for the micro-integration of optical devices. As early as 1996, Davis et al. first used a focusing femtosecond laser in several optical glasses to generate corresponding refractive index change by excitation of nonlinear effects in the focus area (multi-photon absorption, tunneling ionization, avalanche ionization, etc.) and realized three-dimensional modification on micron scale [1]. Low-loss, nondiffractive WGs can be rapidly fabricated with the technology, and the fabrication and integration of active and passive micro-optical devices can be realized in several optical materials. Compared with other WGs preparation technologies, such as epitaxial layer deposition [2], ion/proton exchange [3], ion implantation/irradiation [4], and metal ion diffusion [5], femtosecond laser direct writing is a more efficient and flexible three-dimensional high-precision micro-machining technology [6]. The high-temperature stability of WGs directly written by femtosecond laser largely depends on laser manufacturing parameters and the type of structural modification in the target material [7]. Before that, Yb-doped phosphate glass WGs directly written by femtosecond laser occurred self-annealing in the modified area [8]. These WGs are fabricated by femtosecond pulses with a kHz repetition rate without thermal influence, causing a positive refractive index change. In contrast, the pulse with a high repetition rate will lead to a negative refractive index change in the focus area [9], which shows significant high-temperature stability. In addition, the femtosecond radially polarized vortex laser with a repetition rate of MHz has the advantages of compact focusing spot, high precision, and high writing efficiency. It can inscribe high-quality waveguides inside the crystal, and the vortex retarder (VR) is the critical component to obtain the radially polarized vortex beam.

Waveguide lasers can realize low-loss single-mode coupling propagation and are an ideal laser light source for all-optical network integration [10]. Compared with solid-state lasers, the higher photon density in the waveguide reduces the pump power threshold of laser oscillation and improves the slope efficiency of laser output [11]. Solid-state waveguide lasers have more compact structures and smaller beam divergence angles than multimode fiber lasers. In addition to the CW waveguide laser generation with narrow linewidth [12], [13], mode-locked waveguide lasers have also attracted extensive research interests recently due to their ability to generate extremely high repetition rate pulses [14]. Waveguide lasers have been proven in various rare earth-doped glasses and crystals, which cover a broad-spectrum range [15]. Ytterbium-doped mediums in 1 μm band provide high absorption, emission cross sections, and low quantum defects, making them an ideal choice for direct laser diode (LD) pumping. So far, waveguide lasers have been experimentally demonstrated by Yb-doped crystals such as Yb:GdAl_3_(BO_3_)_4_, Yb:KYW, and Yb:LuYSiO_5_ [16], [17], [18]. Especially, rare earth ion-doped fluoride crystals have low phonon energy, high re-solubility, low nonradiative transition probability, long-life metastable state, and good transparency in the infrared region [19]. Due to these unique physical and chemical properties, fluoride has attracted attention in the infrared photonics field. Among them, Yb:CaF_2_ crystal has many advantages, such as long emission life, high thermal conductivity, and low nonlinear refractive index, making it a very competitive laser material [20]. In addition, the unique fluorite structure of Yb:CaF_2_ crystal combines with the advantages of oxide crystals and glasses [21], which can effectively reduce quantum defects in diode-pumped systems and avoid destructive processes such as excited state absorption or energy transfer.

The practical methods to obtain short pulse waveguide laser output can be divided into Q-switching and mode-locked technology, and the Q-switching technology includes active Q-switching and passive Q-switching [22]. Because of its small size and easy integration with the waveguide, passive Q-switching technology is widely used in waveguide Q-switching [23]. Passive Q-switching primarily involves inserting materials with nonlinear saturable effects in the resonant cavity [22]. As a novel graphene-like two-dimensional material in transition metal sulfides (TMDs), ReS_2(1−*x*)_Se_2*x*_ has excellent potential in photonic and optoelectronic applications due to its fragile interlayer coupling force and intrinsic in-plane anisotropic physical properties [24]. Compared with binary TMDs, ternary TMDs ReS_2(1−*x*)_Se_2*x*_ has a multi-degree of freedom of band regulation [25]. The material’s band gap can be adjusted by changing the proportion of two elements, which provides a new scheme for improving its optical and electronic properties. Based on the saturable absorption effect of ternary TMDs, ReS_0.8_Se_1.2_ is selected as a saturable absorber (SA) to achieve stable Q-switched laser output through the waveguide resonator.

So far, there are few reports about Yb:CaF_2_ waveguide lasers. Petit et al. reported that Yb:CaF_2_ ridge WGs were prepared by ion implantation [26]. Loiko et al. reported that plane waveguide laser was realized in Yb:CaF_2_ crystal. The CW waveguide laser output with a maximum output power of 82.8 mW and a slope efficiency of 6.9 % was obtained at 2 % output coupler (OC) [27]. Compared to the above work, we use more efficient femtosecond laser direct writing technology to induce WGs in crystalline materials rapidly. Ren et al. fabricated low-stress cladding WGs in Yb,Na:CaF_2_ crystal by using ultra-fast direct laser writing technology and two-layer graphene as a SA. Under 946 nm pumping, these WGs realized low pump power threshold CW and Q-switched laser oscillation, ranging from 49.2 kHz (104.8 kHz) to 252.0 kHz (269.3 kHz) at TE (TM) polarization [28]. However, to the best of our knowledge, the single-wavelength low-stress and low propagation loss cladding WGs waveguide lasers in Yb:CaF_2_ crystal directly written by the MHz repetition rate femtosecond radically polarized vortex laser have not been reported.

In this paper, low-stress cladding WGs are fabricated by a focused MHz repetition rate femtosecond radically polarized vortex laser in Yb:CaF_2_ crystal. At 976 nm, these WGs all realize low threshold single-wavelength CW laser oscillations. The ternary TMDs ReS_0.8_Se_1.2_ is used as a SA to realize the Q-switched pulsed waveguide laser output for the first time.

## 2 Experimental methods

Before the experiment, a 5.0 at.% Yb:CaF_2_ crystal was cut and polished to obtain a size of 4 (*x*-axis) × 3 (*y*-axis) × 3 (*z*-axis) mm^3^ sample. We use a homemade all-fiber laser amplifier for the laser direct writing system to generate horizontal linear polarized pulses with a center wavelength of 1030 nm, a pulse duration of 220 fs, and a repetition rate of 1 MHz. Using an infrared antireflection objective lens (50×, NA = 0.65, Olympus), fixing the sample on a translation table (H101A Range), which is connected to a micro-scale controller (ProScan III), and focusing the laser at 130 μm below the surface (4 × 3 mm^2^) of the Yb:CaF_2_ crystal as the initial depth. The scanning area in the front surface (3 × 3 mm^2^) of Yb:CaF_2_ crystal is equally divided by parallel laser scanning tracks, in which the number of laser scanning tracks is *N* and the equal angle size is “*φ*.” The formula is expressed as follows:

(1) $$\phi =\frac{2\pi }{N}$$

**Figure 1:**
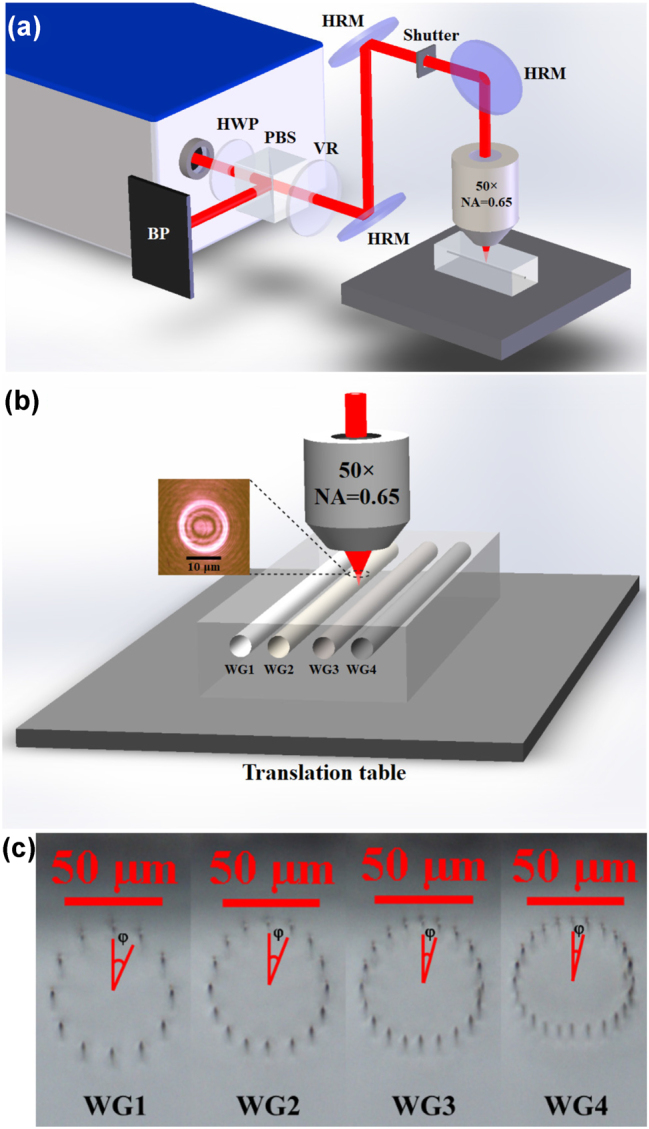
Cladding waveguide processing system diagram and waveguide end face micrograph. (a) The diagram of an experimental device for femtosecond radially polarized vortex laser direct writing Yb:CaF_2_ crystal cladding WGs. HWP: half-wave plate, PBS: polarizing beam splitter, VR: vortex retarder, HRM: high reflectivity mirror, BP: blocking plate. (b) The schematic diagram of cladding WGs fabricated by femtosecond radially polarized vortex laser. The illustration shows the profile of a femtosecond radially polarized vortex beam focused by a NA = 0.65 focusing objective at approximately 10 μm above the focal plane (upper surface of Yb: CaF_2_ crystal). (c) Bright-field microscopy images of cladding WGs end surfaces.

Figure 1(a) shows the experimental device for femtosecond radially polarized vortex laser direct writing Yb:CaF_2_ crystal cladding WGs. Half-wave plate (HWP) and a polarizing beam splitter (PBS) are used to adjust the average laser power deposited on the sample, and a vortex retarder (VR) converts fundamental mode Gaussian beams into Laguerre–Gauss beams. Figure 1(b) shows the principle of cladding WGs fabricated by a femtosecond radially polarized vortex laser. Setting translation table movement speed to 200 μm/s and different pulse energies are adjusted for different focusing depths. That is, when the focus depth is deeper than 115 μm, the pulse energy is adjusted to 0.48 μJ; when the focus depth is less than 77 μm, the pulse energy is adjusted to 0.42 μJ; and when another focusing depth is used, the pulse energy is adjusted to 0.45 μJ. The diameter of all circular cladding WGs is 60 μm, and the numbers of laser scanning tracks are 12 (WG1), 16 (WG2), 20 (WG3), and 24 (WG4), respectively. After the laser crystal is processed, we clean the end surfaces of WGs to reduce scattering loss.

**Figure 2:**
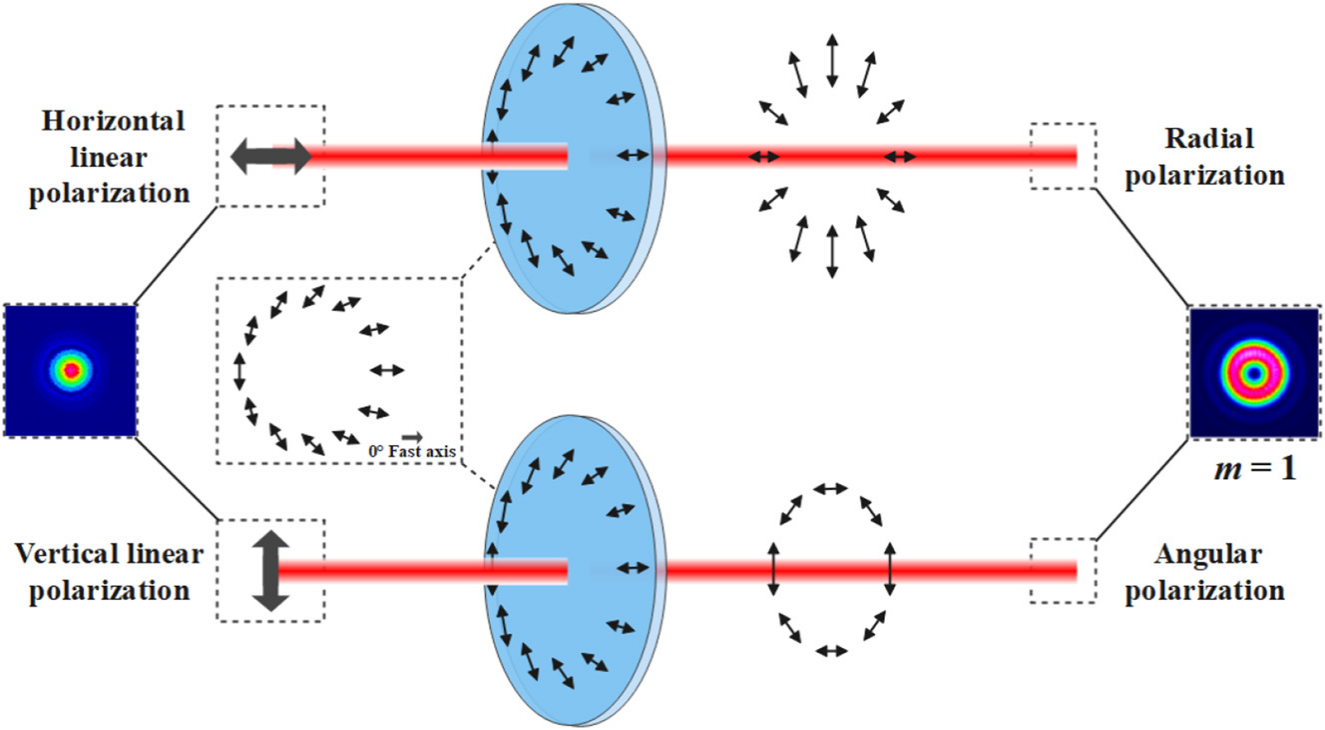
The diagram of radially polarized vortex beam generation.

The VR is a polarization element and single wavelength device related to the incident light’s polarization state. The VR can generate a polarized vortex beam when the incident beam is linear polarization. There are two exceptional cases: if the polarization direction of the incident polarized beam is parallel to the 0° fast axis of the VR, and the output beam is radial polarization, i.e., the direction of polarization at each point of the beam cross section is parallel to the radial direction; if the polarization direction of the incident polarized light is vertical to the 0° fast axis of the VR, and the output beam is angular polarization, i.e., the direction of polarization at each point of the beam cross section is vertical to the radial direction, as shown in Figure 2. The order m is the topological charge. The smaller the value of m, the smaller the size of the central hole of the outgoing beam [29]. The VR (*m* = 1, LBTEK) with a working wavelength of 1030 nm is selected to generate a femtosecond radially polarized vortex laser. Adjusting the VR to meet the condition that the polarization direction of incident pulses is parallel to the 0° fast axis of the VR, so the radially polarized Laguerre–Gauss vortex beam output can be achieved. As shown in Figure 1(b), the spot profile of the femtosecond radially polarized vortex laser is measured at about 10 μm above the upper surface of Yb: CaF_2_ crystal using CCD (charge-coupled device), and its center size is about 10 μm. Figure 1(b) is actually only intended to clearly display the spot contour of the femtosecond radially polarized vortex beam. In fact, the diameter of the focal spot focused at 100 μm below the upper surface of the Yb:CaF_2_ crystal is about 1.3 μm. The focal light field of the femtosecond radially polarized vortex beam focused by a high numerical aperture objective lens comprises a solid and narrow longitudinal component and a radial component, and the longitudinal component is dominant. Compared with linearly polarized light and circularly polarized light, the intensity peak of the longitudinal component of radially polarized light is sharper and more compact. Reflected in the focal spot’s size, the focal spot of radially polarized light is smaller than that of linearly polarized and circularly polarized light [30]. Using femtosecond radially polarized light in the experiment to write crystal waveguides can get a better writing effect.

**Figure 3:**
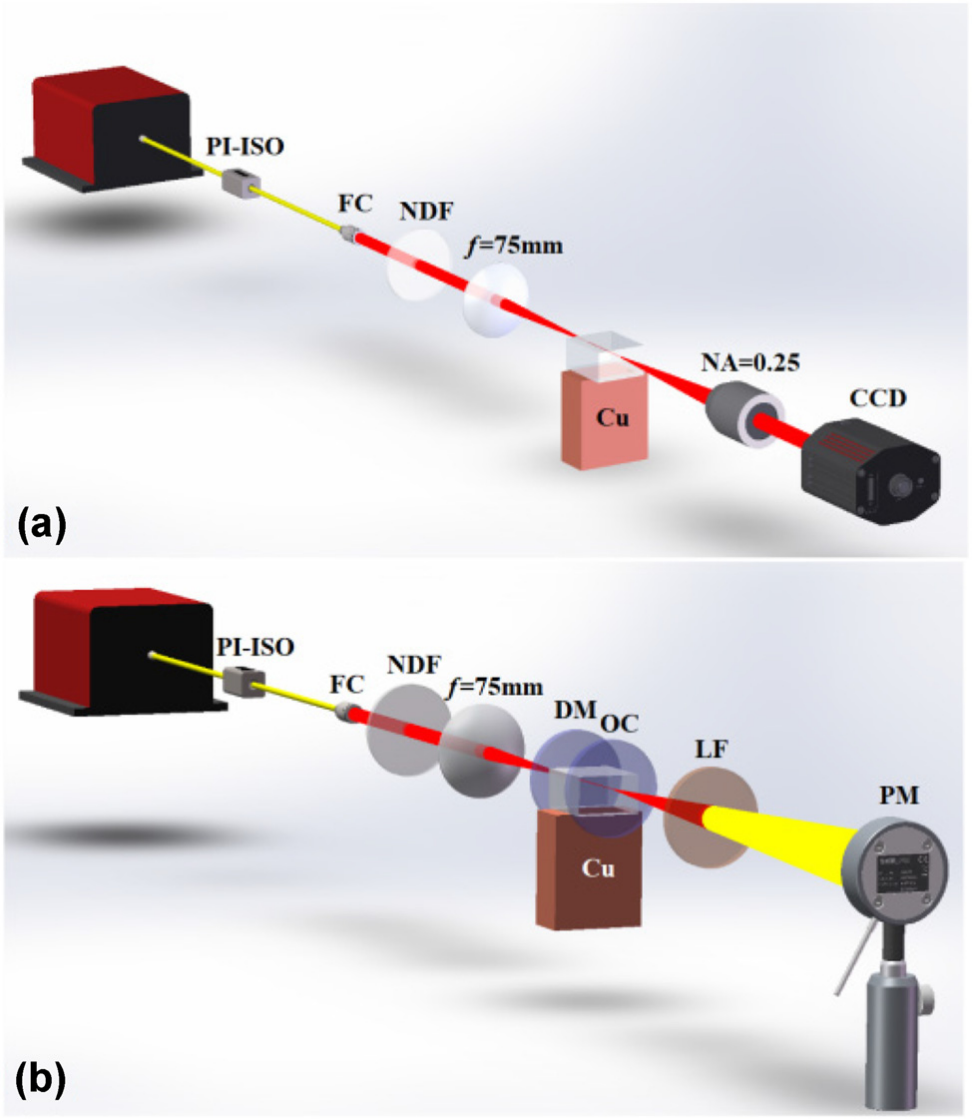
End coupling system diagram and waveguide laser system diagram. (a) The diagram of the experimental setup of the waveguide end-coupling system. PI-ISO: polarization-independent isolator, FC: fiber collimator, NDF: neutral density filter, CCD: charge-coupled device. (b) The diagram of experimental waveguide laser setup. DM: dichroic mirror, OC: output coupler, LF: long pass filter, PM: power meter.

We measure the properties of different circular cladding WGs at pump wavelength by using an end-coupling system, as shown in Figure 3(a). The experiments of propagation loss measurement and CW laser generation are carried out by using 976 nm single mode LD as a pump source. The pump source is connected with a polarization-independent isolator (PI-ISO) and a fiber collimator (FC) through single-mode fiber (SMF), and the waist spot radius is expanded to 1.05 mm. The neutral density filter is adjusted to control input pump power, and then the front surfaces of WGs are coupled through a plano-convex lens (PCL) with a focal length of 75 mm. It is worth noting that the radius of the focused waist spot is 21 μm, which can match the mode field of cladding WGs well and improve the coupling efficiency of pump lasers. The crystal is stuck by heat dissipation silicone grease on a copper sheet fixed to a 6-axis translation table, and the translation table is moved to optimize the coupling conditions of cladding WGs. The output beams are received by an objective microscope lens (10×, NA = 0.25, Nikon) on the back surface of WGs, and the near-field profile images are recorded by an infrared charge-coupled device (CCD, Contour IR Digital).

After measuring the propagation loss of cladding WGs, we use the PCL as a coupling lens. A compact resonant cavity comprises a dichroic mirror (DM) with a curvature radius of *R* = 200 mm and a planar output coupler (OC). The DM has 98 % transmissivity in the 940–980 nm range and over 99.5 % reflectivity in the range of 1010–1100 nm. The OC has 90 % reflectivity near the center wavelength of 976 nm and 2 % transmissivity in the range of 1000–1100 nm. Notably, the planar OC must be placed close to the back surface of Yb:CaF_2_ to reduce geometric deflection loss in a resonant cavity. Then, the unabsorbed pump laser of the resonant cavity is filtered through a planar long pass filter (LF). Finally, an infrared charge-coupled device (CCD, Duam) and power meter (PM, Nova II display) are placed to record the parameters of waveguide lasers, as shown in Figure 3(b).

**Figure 4:**
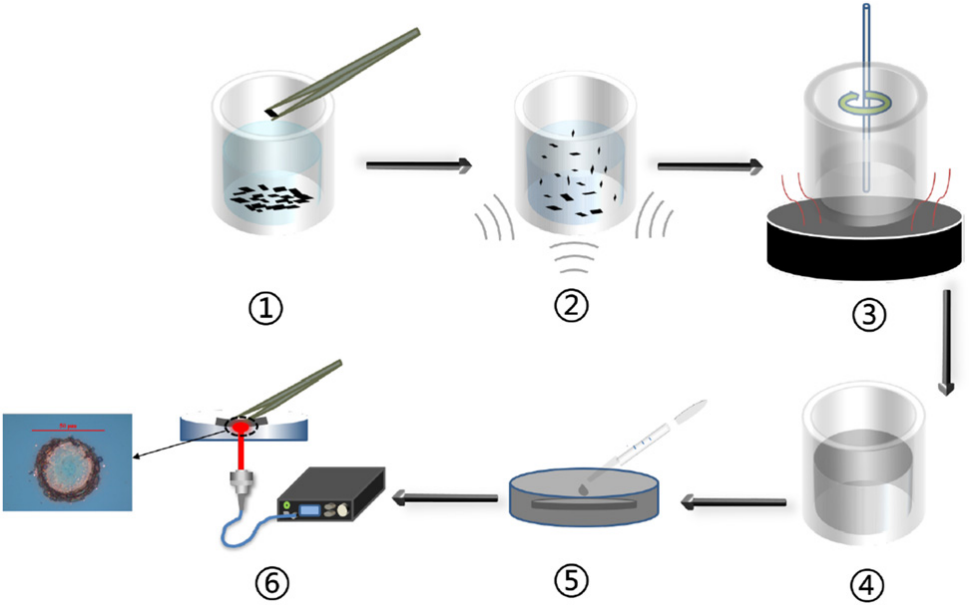
Preparation of the ReS_0.8_Se_1.2_ SAM.

The ReS_0.8_Se_1.2_ thin film is prepared by a polyvinyl alcohol (PVA) film-forming agent. The preparation process is shown in Figure 4. Firstly, PVA powder is dissolved in a certain amount of plasma water with a concentration of 5 % wt. and thoroughly stirred. After the PVA is completely dissolved, we take 5 ml PVA solution and add some ReS_0.8_Se_1.2_ powder to the PVA solvent. Then, conduct ultrasonic treatment for 2 h, thoroughly stir to make it mix evenly, transfer it to the glass substrate, and place it in the drying oven for natural evaporation. After 24 h, there is a layer of ReS_0.8_Se_1.2_-PVA thin film on the glass substrate. Then, we cut the prepared ReS_0.8_Se_1.2_-PVA film into 5 × 5 mm^2^ and paste it on the reflective surface (concave) of DM. After laser irradiation, the SAM based on ReS_0.8_Se_1.2_ is finally obtained.

**Figure 5:**
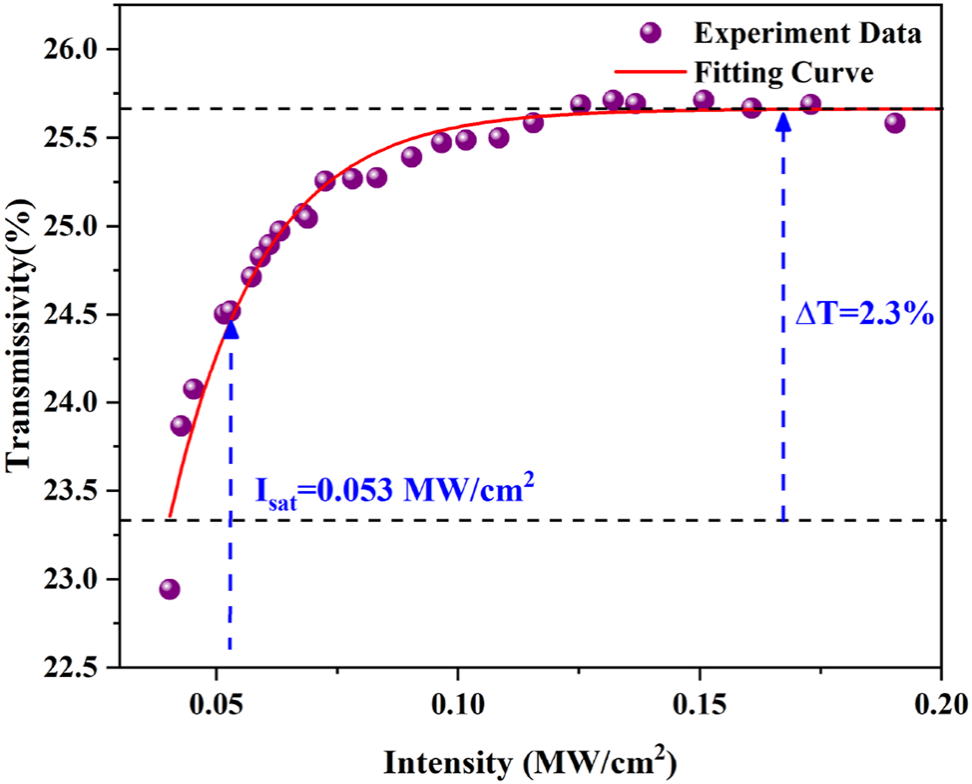
The ReS_0.8_Se_1.2_-SA nonlinear optical absorption curve.

The nonlinear saturable absorption characteristics of its ReS_0.8_Se_1.2_-PVA single-layer thin film are measured using a twin-detector technique method. The laser source uses a nonlinear polarization rotation (NPR) mode-locked ytterbium-doped fiber laser with a repetition rate of 33.9 MHz, a center wavelength of 1030 nm, and a pulse width of 200 fs. After fitting the experimental data, the saturable absorption curve of the material can be obtained. As shown in Figure 5, the modulation depth of ReS_0.8_Se_1.2_ is 2.3 %, and the saturable absorption intensity is 0.053 MW/cm^2^.

**Figure 6:**
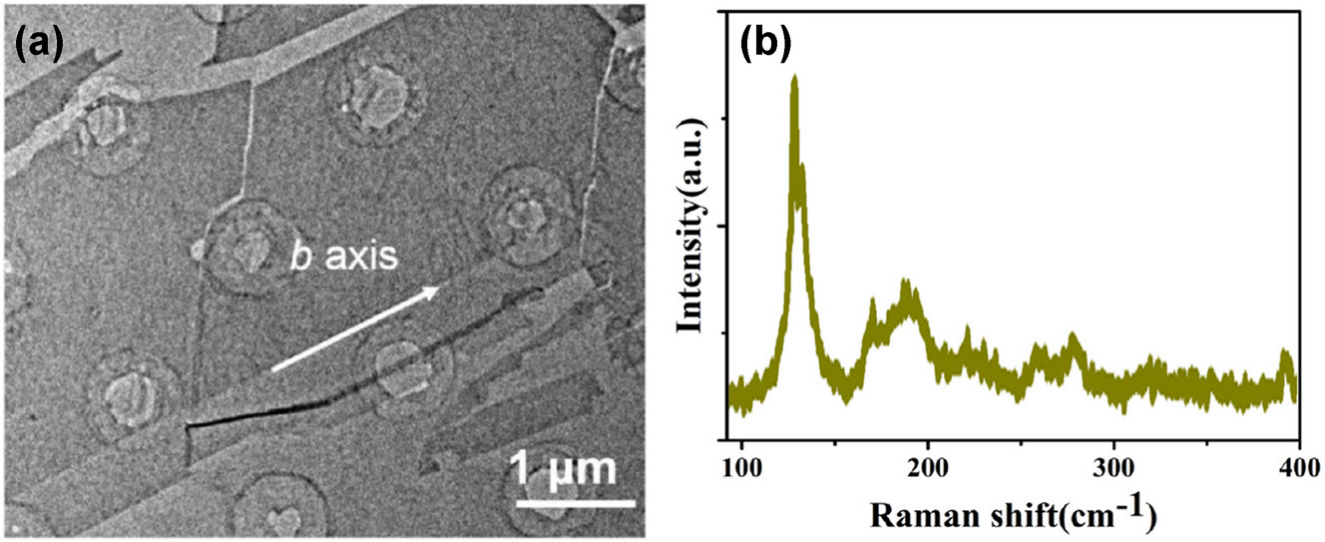
Characteristics of ReS_0.8_Se_1.2_. (a) TEM image of ReS_0.8_Se_1.2_. (b) Raman spectrum [24].

The ReS_0.8_Se_1.2_ used in this work is from the previously published work [24]. The exact preparation method and material characterization of ReS_0.8_Se_1.2_ are shown in Reference [24]. Through X-ray energy dispersive spectroscopy (EDS), it can be concluded that the elemental strength ratio of S element and Se element is 0.41:0.59. As shown in Figure 6(a), from transmission electron microscopy (TEM), we can see the homogeneous spatial distribution of Re, S, and Se elements. ReS_2(1−*x*)_Se_2*x*_ have theoretically 18 Raman active peaks. The mode will be less than this number in the experiment due to the lapping of some Raman modes. The Raman spectrum of ReS_0.8_Se_1.2_ is shown in Figure 6(b). Re atoms vibration peak was at lower-frequency positions (100 cm^−1^–160cm^−1^). S and Se atoms’ vibration peaks were at high-frequency positions (160 cm^−1^–400 cm^−1^). The Raman modes were similar to the ReS_1.02_S_0.98_ we used in previous work [25]. The difference is that the modes’ Raman frequency has a redshift, which is related to the content of the Se element.

## 3 Results and discussions

### 3.1 Waveguide properties

**Figure 7:**
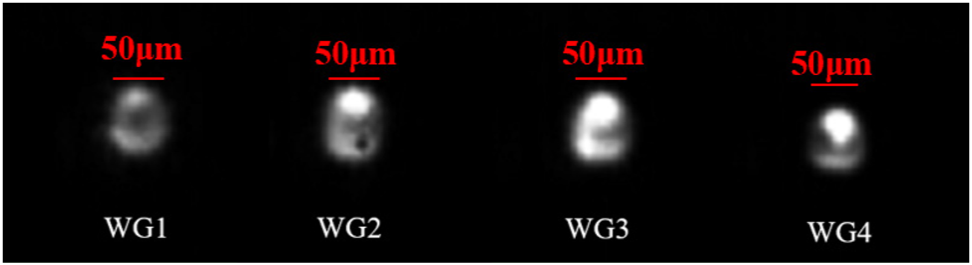
The image of cladding WGs output near-field profiles (pumped light [*λ* = 976 nm] as the test wavelength).

The Laguerre–Gauss beam is focused in Yb:CaF_2_ crystal, a higher spatial resolution, and strong nonlinear effect occur in its focal region, while the translation table moves back and forth at a uniform speed along the *x*-axis of the crystal, causing the focal region to translate within the crystal and generating laser scanning tracks parallel to each other. We use different incident powers for laser scanning tracks of different depths to obtain a uniform radial distribution of significant refractive index change in low-stress circular cladding WGs. Figure 1(c) shows bright-field microscopy images of a series of circular cladding WGs, with a length of 4 mm. Due to the elastic-optical effect, the stress affects the local change of the refractive index in the waveguide region, resulting in a slight refractive index difference inside and outside laser scanning tracks, where the maximum refractive index change occurs in WG4. Due to the extremely short space between the scanning tracks on both sides of WG4, laser focal area scans overlap, resulting in a small crack in this region during the direct writing process, which also greatly increases propagation loss of the waveguide and affects the laser properties of the waveguide laser in subsequent laser experiments. Figure 7 shows the near-field pattern of the pump spot emitted after coupling different waveguides through the FCL with *f* = 75 mm. It can be obviously seen that the pump light mode has very clear boundaries, indicating that the 976 nm pump light is well confined within the waveguide. Table 1 shows the measured Δ*n*, NA, and *α*_*P*_ of different waveguides.

**Table 1:** The Δ*n*, NA, and *α*_*P*_ of cladding WGs.

	WG1	WG2	WG3	WG4
Δ*n*	1.9 × 10^−4^	2.2 × 10^−4^	2.3 × 10^−4^	2.6 × 10^−4^
NA	0.022	0.024	0.024	0.026
*α*_*P*_ (dB/cm)	1.27	0.85	1.00	1.02

Figure 3(a) shows the experimental setup of the waveguide end-coupling system. The following equation expresses the insertion loss *α*_*S*_:

(2) $$\alpha_{S}\;=\;-\;10\times \mathrm{lg}\left(\frac{P_{\text{out}}}{P_{\text{in}}}\right)$$

where *P*_out_ is the optical power output from the back surface of the crystal and *P*_in_ is the optical power input from the front surface of the crystal [31], [32]. Considering that the incident optical waist radius *ω*_0_ overlaps with the waveguide mode radius *ω*_1_, the normalized coupling factor *C* and coupling loss *α*_*C*_ can be calculated according to the following equation:

(3) $$C={\left(\frac{2\omega_{0}\omega_{1}}{{\omega_{0}}^{2}\;+\;{\omega_{1}}^{2}}\right)}^{2}$$

(4) $$\alpha_{C}\;=\;10\times \text{lg}C$$

The end surface Fresnel reflection loss *α*_*R*_ can be calculated from the following equation:

(5) $$\alpha_{R}\;=\;-\;10\times \mathrm{lg}\left(1-R\right)$$

where *R* is reflectivity of the material end surface, and it is expressed by the following equation:

(6) $$R={\left(\frac{n_{1}\;-\;n_{0}}{n_{1}\;+\;n_{0}}\right)}^{2}$$

where *n*_1_ and *n*_0_ are the refractive indices of the Yb:CaF_2_ crystal and air. Considering the strong absorption of Yb:CaF_2_ crystal at 976 nm, we measure and calculate the absorption coefficient *α*_*A*_ of the crystal by control experiments, which is expressed by the following equation:

(7) $$\alpha_{A}\;=\;\frac{\alpha_{S}\;-\;2\alpha_{R}}{2Z_{R}}$$

In the control experiment, the incident waist spot radius of the pump laser should be consistent with the waveguide mode radius *ω*_1_. *Z*_*R*_ is the Rayleigh distance of focused pump laser, and *L* is the length of the cladding waveguide. The following equation calculates the propagation loss *α*_*P*_:

(8) $$\alpha_{P}\;=\;\frac{\alpha_{S}\;-\;\alpha_{C}\;-\;2\alpha_{R}}{L}\;-\;\alpha_{A}$$

We estimate the refractive index change (Δ*n*) of cladding WGs by the following equation [33]:

(9) $$\Delta n=\frac{\sin{\theta_{m}}^{2}}{2n_{1}}$$

where *θ*_*m*_ is the maximum exit angle.

### 3.2 CW waveguide laser

**Figure 8:**
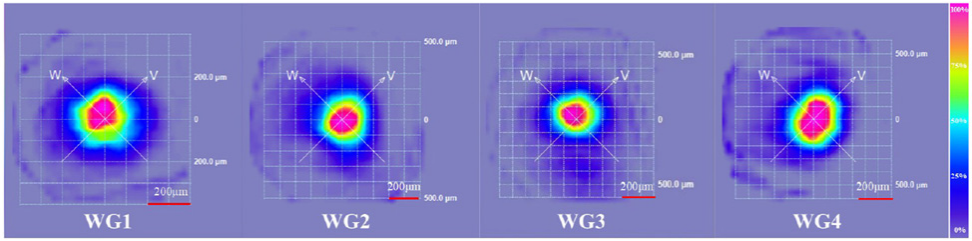
The image of waveguide laser near-field modes.

**Figure 9:**
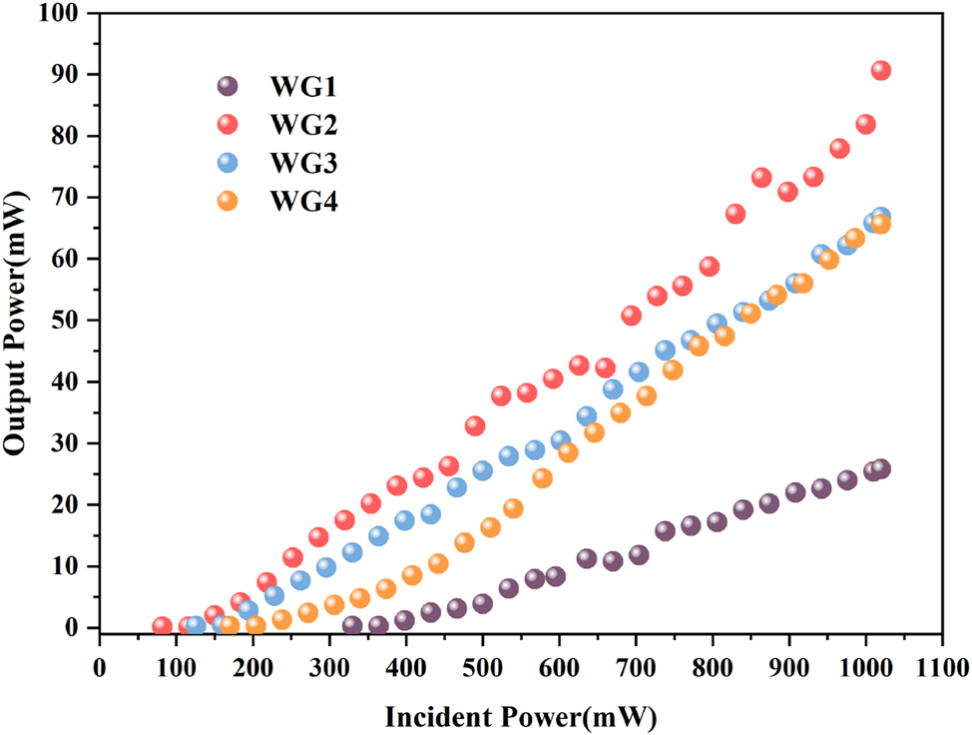
Variation of cladding waveguide lasers output power with incident pump power.

It is found that all four cladding WGs can support CW laser oscillations, and Figure 8 shows the near-field modes of waveguide lasers, respectively. Figure 9 shows the variation of output power with input power for cladding WGs. The WG2 demonstrates the best laser performance at the same pump wavelength and coupling condition. The CW waveguide laser threshold is as low as 116 mW, and slope efficiency reaches 9.4 %. The other waveguide laser thresholds correspond to 364 mW for WG1, 183 mW for WG3, and 233 mW for WG4, with slope efficiencies of 4.0 %, 7.6 %, and 8.5 %, respectively. Compared to the threshold of solid-state laser, which is close to 1 W, waveguide structure can reduce laser threshold while improving compactness. The WG2 has the lowest propagation loss and supports single-mode propagation, making it meet the lower threshold properties. The laser threshold of other WGs is also mainly determined by their propagation loss. For WG1, a few laser scanning tracks enclose a waveguide core layer that cannot wholly confine the diffraction-free propagation of the optical field, resulting in a significant energy spread. It can be seen from cladding waveguide laser modes. The WG4 with the densest laser scanning tracks has a significant output waveguide laser mode distortion because overlapping scanning on both sides of the waveguide accumulates severe lattice damage, resulting in output laser mode appearing off-core, and this waveguide mode distortion also makes the further increase of CW laser threshold. Single-mode waveguide propagation ensures a good mode match between the pump and laser modes, reducing the threshold and increasing laser slope efficiency. However, the single-mode property reduces the laser divergence angle while limiting the energy transfer in the waveguide compared to the few-mode waveguide lasers [34]. Under the same coupling conditions, the fluorescence emission intensities correlate significantly with the propagation loss of different cladding WGs, so optimizing the propagation loss of the WGs is an essential aspect of waveguide laser generation.

**Figure 10:**
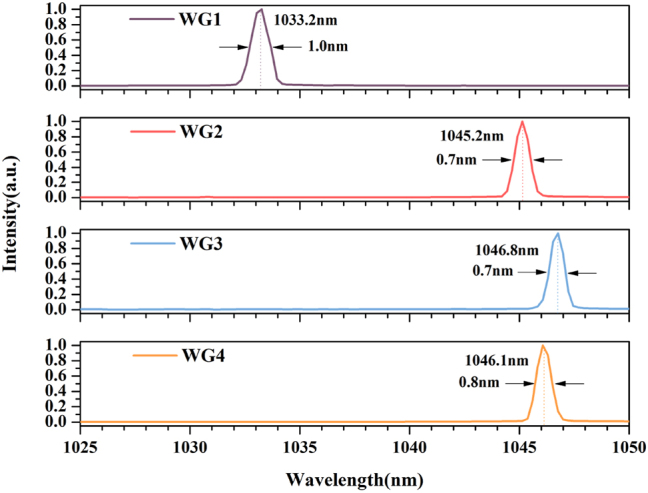
The image of laser spectra of cladding WGs (normalized).

With increasing pump power and fine adjusting of DM, the single-wavelength CW laser is generated. Subsequently, we used a spectrometer (Avaspec-2048) with an accuracy of 0.7 nm to measure its continuous waveguide laser spectrum. For WG1, when the incident pump power is over 364 mW, the CW laser is generated at a center wavelength of 1033.2 nm. When incident pump power is larger than 116 mW, the CW laser with the center wavelength at 1045.2 nm is generated in WG2. For WG3, when the incident pump power exceeds 183 mW, the CW laser is generated at a center wavelength of 1046.8 nm. When the incident pump power is larger than 233 mW, the CW laser is generated at a center wavelength of 1046.1 nm in WG4. Figure 10 shows the spectra of waveguide lasers (normalized), where the full width at half maximum (FWHM) of 1 nm, 0.7 nm, 0.7 nm, and 0.8 nm, respectively. In this work, the Yb:CaF_2_ crystal obtained single-wavelength CW laser output under the 976 nm pump source. Compared to the work [27], we achieve higher slope efficiency (9.4 %) and narrower FWHM (0.7 nm) with a shorter cavity length (4 mm), using the same 2 % OC. Single-wavelength laser operation is mainly explained by the Yb^3+^ three energy levels system model and short cavities [35]. In the Yb^3+^ emission bandwidth, a stable single-wavelength oscillation is achieved because the waveguide structure can limit the optical field to generate a high laser gain over the length of the crystal, and an extremely short resonant cavity enables only one longitudinal mode to exist in the emission bandwidth. The generation of linewidth is mainly influenced by the spontaneous radiation of excited state atoms or ions, phase noise, and external factors such as mechanical vibration and temperature shift. The smaller the linewidth value, the higher the purity of the spectrum, i.e., the better the monochromaticity of the laser. The waveguide laser usually has tiny phase or frequency noise and relative intensity noise, while the particular structure of the single-mode waveguide ensures that no high-order mode can oscillate in the resonant cavity. Table 2 shows the performance parameters of different waveguide lasers.

**Table 2:** The performance parameter of cladding WGs laser.

	WG1	WG2	WG3	WG4
Waveguide laser threshold (mW)	364	116	183	233
Slope efficiency	4.0 %	9.4 %	7.6 %	8.5 %
Central wavelength (nm)	1033.2	1045.2	1046.8	1046.1
FWHM (nm)	1	0.7	0.7	0.8

### 3.3 Q-switched waveguide laser emission by ReS_0.8_Se_1.2_ SA

**Figure 11:**
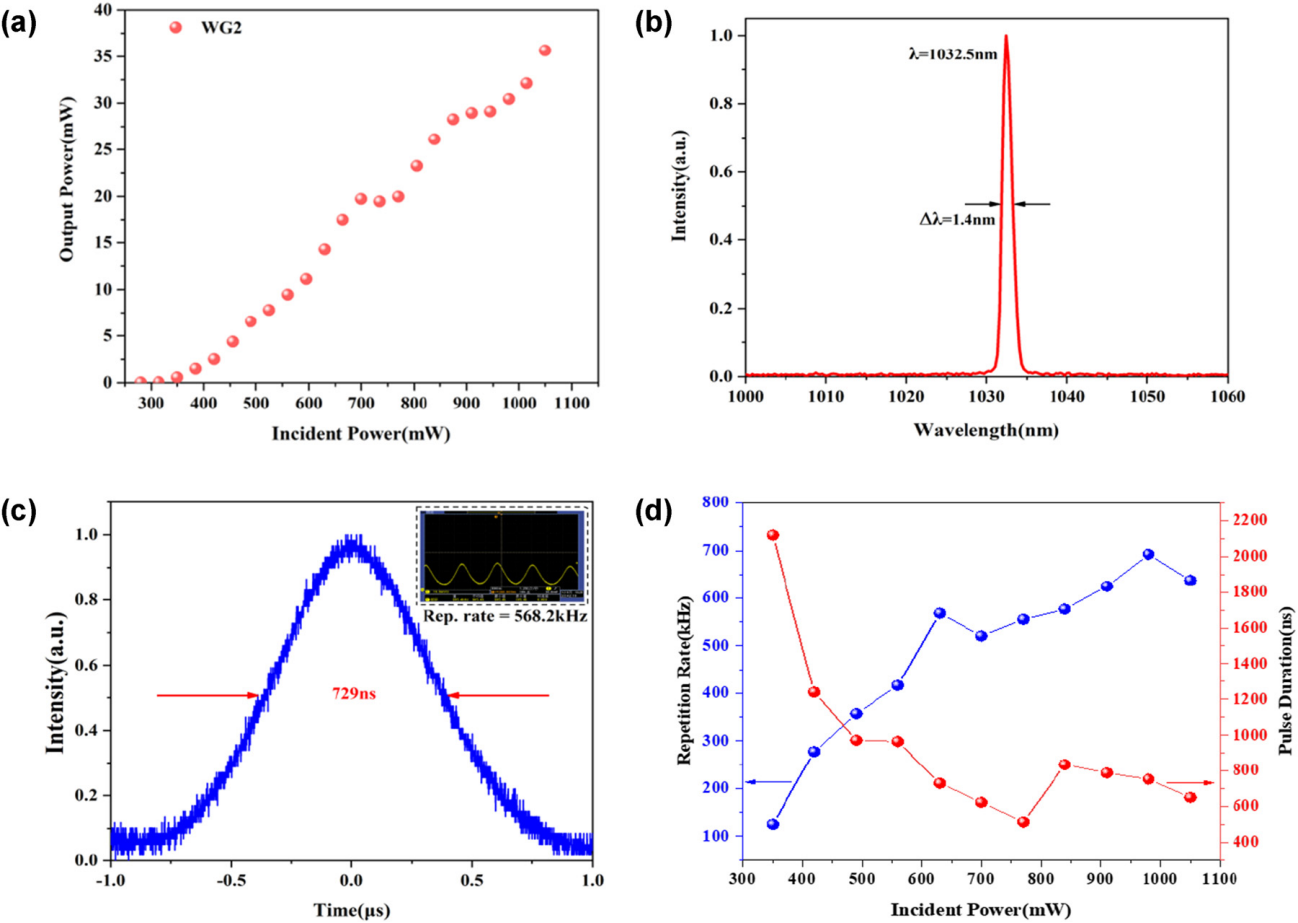
The results of Yb:CaF_2_ Q-switched waveguide laser. (a) The output power of Q-switched waveguide laser varies with incident pump power. (b) The Q-switched waveguide laser spectrum. (c) The single pulse profile with incident pump power of 630 mW. The illustration shows the oscilloscope pulse sequence. (d) The variation of pulse duration and repetition rate of Q-switched waveguide laser with incident pump power.

Since WG2 has the lowest threshold and the highest slope efficiency in the above CW waveguide laser experiment, the Q-switched waveguide laser experiment is carried out with WG2. Using the prepared ReS_0.8_Se_1.2_ saturable absorption mirror as the pump mirror, a waveguide resonator, as shown in Figure 3(b), is built and pumped by the 976 nm single-mode laser to obtain the Q-switched pulse laser output. The threshold of the Q-switched laser is 308 mW. At the highest pump power of 1050 mW, the maximum output power is 35.6 mW. The corresponding slope efficiency is 5.04 %, as shown in Figure 11(a). The spectrum of the output laser is shown in Figure 11(b), with a center wavelength of 1032.5 nm and a FWHM of 1.4 nm. The individual pulse profile is shown in Figure 11(c); it shows the Q-switched pulse train at the input pump power of 630 mW. Figure 11(d) shows the variation of pulse duration and repetition rate with pump power. When the input power is greater than 800 mW, the thermal stability of the saturable absorber is affected, resulting in instability of its Q-switched pulse and sudden changes in its repetition rate and pulse width. With the pump power increases, the repetition rate can be tunable in the range of 125 kHz–692.5 kHz, with a minimum pulse duration of 513 ns. Compared to the previous work [28], we use ternary TMDs ReS_0.8_Se_1.2_ as a SA to achieve Q-switched pulse output with a larger repetition rate tuning range.

## 4 Conclusions

In summary, we have fabricated a low-stress circular cladding WGs in Yb:CaF_2_ crystal, which is directly written by a homemade MHz repetition rate all-fiber femtosecond radially polarized vortex laser system. Single-wavelength CW waveguide laser oscillation is achieved for all these waveguide structures under 976 nm pump wavelength. A saturable absorber mirror is prepared by depositing ReS_0.8_Se_1.2_ on the reflective surface of the DM, and the Q-switched pulse with a tunable repetition rate in the range of 125–692.5 kHz and the minimum pulse duration of 513 ns is achieved. The excellent physical and chemical properties of calcium fluoride material and the excellent nonlinear optical properties and pulse modulation characteristics of ReS_0.8_Se_1.2_ material make the compact waveguide laser have a broad application prospect in the fields of micro-optics and integrated photonics.
